# Mirau-based line-field confocal optical coherence tomography for three-dimensional high-resolution skin imaging

**DOI:** 10.1117/1.JBO.27.8.086002

**Published:** 2022-08-13

**Authors:** Weikai Xue, Jonas Ogien, Pavel Bulkin, Anne-Lise Coutrot, Arnaud Dubois

**Affiliations:** a Université Paris-Saclay, Institut d’Optique Graduate School, CNRS, Laboratoire Charles Fabry, Palaiseau, France; b DAMAE Medical, Paris, France; c LPICM, CNRS, Ecole Polytechnique, Institut Polytechnique de Paris, Palaiseau, France

**Keywords:** optical coherence tomography, optical imaging, confocal microscopy, dermatology, skin cancer

## Abstract

**Significance:**

Line-field confocal optical coherence tomography (LC-OCT) is a recently introduced high-resolution imaging modality based on a combination of low-coherence optical interferometry and reflectance confocal optical microscopy with line illumination and line detection. Capable of producing three-dimensional (3D) images of the skin with cellular resolution, *in vivo*, LC-OCT has been mainly applied in dermatology and dermo-cosmetology. The LC-OCT devices capable of acquiring 3D images reported so far are based on a Linnik interferometer using two identical microscope objectives. In this configuration, LC-OCT cannot be designed to be a very compact and light device, and the image acquisition speed is limited.

**Aim:**

The objective of this work was to develop a more compact and lighter LC-OCT device that is capable of acquiring images faster without significant degradation of the resolution and with optimized detection sensitivity.

**Approach:**

We developed an LC-OCT device based on a Mirau interferometer using a single objective. Dynamic adjustment of the camera frequency during the depth scan is implemented, using a faster camera and a more powerful light source. The reflectivity of the beam-splitter in the Mirau interferometer was optimized to maximize the detection sensitivity. A galvanometer scanner was incorporated into the device for scanning the illumination line laterally. A stack of adjacent B-scans, constituting a 3D image, can thus be acquired.

**Results:**

The device is able to acquire and display B-scans at 17 fps. 3D images with a quasi-isotropic resolution of ∼1.5  μm (1.3, 1.9, and 1.1  μm in the x,y, and z directions, respectively) over a field of 940  μm×600  μm×350  μm (x×y×z) can be obtained. 3D imaging of human skin at cellular resolution, *in vivo*, is reported.

**Conclusions:**

The acquisition rate of the B-scans, at 17 fps, is unprecedented in LC-OCT. Compared with the conventional LC-OCT devices based on a Linnik interferometer, the reported Mirau-based LC-OCT device can acquire B-scans ∼2 times faster. With potential advantages in terms of compactness and weight, a Mirau-based device could easily be integrated into a smaller and lighter handheld probe for use by dermatologists in their daily medical practice.

## 1 Introduction

Line-field confocal optical coherence tomography (LC-OCT) is a high-resolution imaging modality based on a combination of low-coherence optical interferometry and reflectance confocal optical microscopy.^1^ Capable of producing images of the skin with cellular resolution, *in vivo*, LC-OCT has been mainly applied in dermatology^2^^–^^9^ and dermo-cosmetology.^10^ The LC-OCT technology is derived from time-domain optical coherence tomography (TD-OCT) with line illumination and line detection rather than point scanning illumination and point detection.^11^^,^^12^ By acquiring multiple A-scans in parallel, a vertical section image (a B-scan) is obtained in LC-OCT by scanning only in the depth direction. A B-scan can therefore be obtained with a depth scan that is slower than in the case of point-scanning TD-OCT, while maintaining a similar acquisition time of the whole image. By depth scanning at a frequency on the order of 10 Hz, a high numerical aperture (NA) microscope objective can be dynamically focused for imaging with high lateral resolution. A broadband supercontinuum laser is employed as a light source with ultrashort temporal coherence for imaging with high axial resolution. A quasi-isotropic image resolution on the order of 1  μm is finally achieved with LC-OCT. In addition, even if not as efficient as in point-scanning confocal microscopy, the linear sensor of the camera used in LC-OCT, which is optically conjugated with the illumination line, acts as a confocal slit that filters most of the out-of-focus parasitic light. The overall signal-to-noise ratio (SNR) is therefore high enough to allow for real-time *in vivo* imaging at a depth up to ∼400  μm in highly scattering tissues like human skin.^2^^,^^13^^,^^14^

The LC-OCT devices that can acquire three-dimensional (3D) images reported so far have been based on a Linnik interferometer using two identical microscope objectives.^13^^,^^14^ In this configuration, the devices could not be designed to be very compact and light, especially when two motorized displacements were used.^1^ In the most compact configuration using a single displacement, the entire Linnik interferometer was moved.^2^^,^^13^^,^^14^ Due to the mass being moved, the inertia limited the acquisition rate of B-scans to 10 Hz. An LC-OCT device based on a Mirau interferometer was recently proposed, with the advantage of being more compact and lighter. B-scans could be acquired at 12 Hz, but only at a fixed location. 3D images could not be obtained with this device.^15^

In this paper, we present a Mirau interferometer-based LC-OCT device that, in addition to acquiring and displaying B-scans at unprecedented speed in LC-OCT, can also acquire 3D images. Cross-sectional images in an arbitrary orientation, including horizontal (*en face*) section images, can be obtained by postprocessing the 3D image. The LC-OCT device, including the specifically designed Mirau interferometer, is described. The performance is reported in terms of spatial resolution and acquisition speed. Images of skin tissue are shown to illustrate the ability of this LC-OCT device to produce 3D images of the skin, *in vivo*.

## 2 Experimental Setup

### 2.1 General Layout

**Fig. 1:**
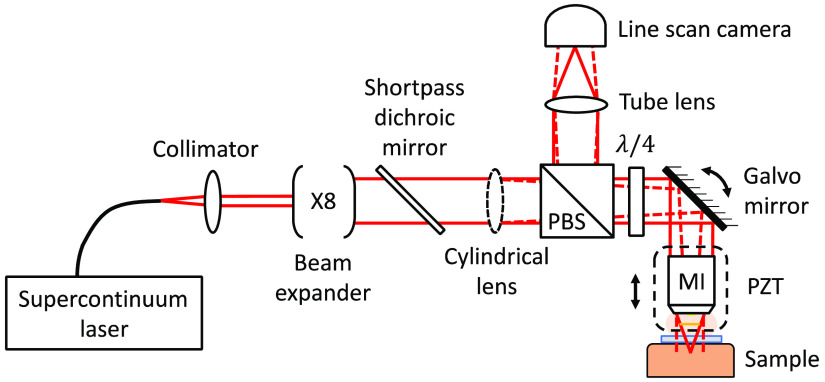
Schematic of the LC-OCT device. PBS, polarizing beam-splitter; λ/4, quarter-wave plate; PZT, piezoelectric-driven linear stage; MI, Mirau interferometer. The red solid lines represent the laser beam in the plane of the figure, and the red dashed lines represent it in an orthogonal plane.

The general experimental setup is shown schematically in Fig. 1. Broadband spatially coherent light emitted by a supercontinuum laser (SMHP-80.2, Leukos) is injected into the setup through a single-mode optical fiber and an achromatic collimator. The collimated Gaussian beam passes through two reflective beam expanders (BEO2R and BEO4R, Thorlabs) to increase its diameter by a factor of 8 ($1/e^{2}$ diameter of 6.4 mm). A shortpass dichroic mirror (DMSP1000, Thorlabs) is placed to avoid illuminating the skin at wavelengths above 1000 nm, which are present in the supercontinuum but are not detected by the CMOS camera used to acquire the images. This optical filter also prevents the detection of the strong pump peak at the 1064-nm wavelength (the effective spectrum is shown in Sec. 4.1). The laser beam passes through a +100-mm focal length cylindrical lens to generate a line of light in the focal plane of the objective. Going through a broadband polarizing beam-splitter linearizes the polarization of the laser beam before it becomes circular due to an achromatic quarter-wave plate (AQWP10M-980, Thorlabs). A galvanometer scanner (6220H, Cambridge Technology) redirects the laser beam in a custom-designed Mirau interferometer to scan the line of light in the focal plane of the objective. The Mirau interferometer is attached to a piezoelectric (PZT)-driven linear stage (P-628.1CD, Physik Instrumente) to scan the line of light in the sample along the axial direction (depth). Light returning from the sample and collected by the objective is once again linearly polarized by the quarter-wave plate, but orthogonally to the incident polarization, so it is totally reflected by the polarizing beam-splitter. The use of the polarizing beam-splitter and the quarter-wave plate reduces the loss of light by a factor of two, compared with the use of a 50:50 beam-splitter alone. The line of light in the focal plane of the objective is conjugated to the sensor of a 2048 pixels CMOS line scan camera (Octoplus, Teledyne e2v) through a +200-mm focal length lens tube (TTL200-B, Thorlabs), which yields a lateral field of view of 940  μm.

### 2.2 Mirau Interferometer

**Fig. 2:**
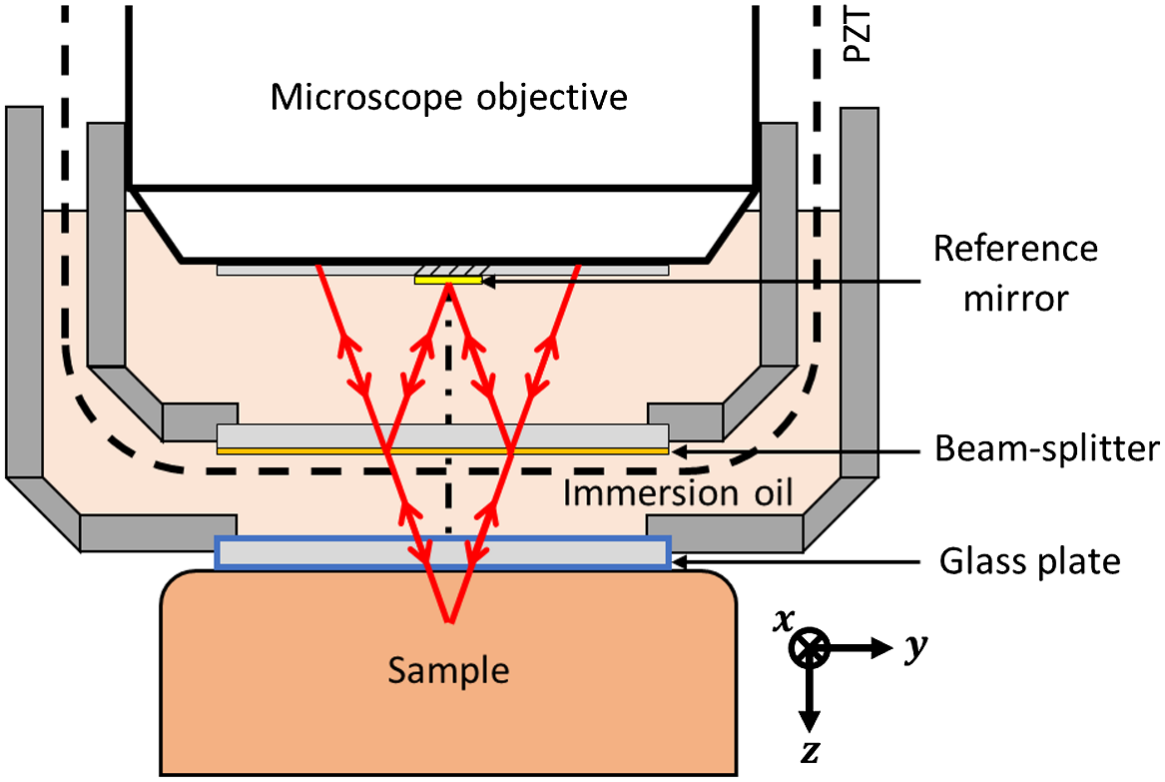
Detailed schematic of the Mirau interferometer. The red lines represent the path followed by the incident light in the plane not affected by the cylindrical lens. The line of light in the focal plane of the objective is parallel to the x-axis.

A detailed illustration of the Mirau interferometer is shown in Fig. 2. It consists of a 20× water-immersion microscope objective with an NA of 0.5 (UMPLFLN 20XW, Olympus), a reference mirror, and a beam-splitter. The reference mirror is a ∼200-nm-thick layer of gold deposited in a rectangular shape (1100  μm×620  μm,x×y) on a 170-μm-thick glass plate glued to the last diopter of the microscope objective using UV glue (Vitralit VBB-1). The reflectivity of the reference mirror at the wavelength of 700 nm is $R_{\mathrm{ref}}=96\%$. The beam-splitter is made of a layer of hydrogenated amorphous silicon (a-SiH) deposited on a 500-μm-thick fused silica glass plate and joined together with the microscope objective via a tank. The thickness of the a-SiH layer was adjusted to 9 nm during the deposition process. The reflectivity of the beam-splitter, measured at $R_{\text{BS}}=11\%$ at the 700-nm wavelength, is thus close to the optimum to maximize the SNR for skin imaging (see the calculations and discussion in Sec. 3.1). The axial position of the beam-splitter is adjusted so that the line of light is focused on the reference mirror. The Mirau interferometer is partially immersed in another tank filled with silicone oil. The bottom of this tank has a 500-μm-thick fused silica window. Depth-scanning is achieved by the PZT-driven linear stage, which translates the microscope objective, the reference mirror, and the beam-splitter together. Compared with the Linnik configuration using a microscope objective in each arm of the interferometer and a cube beam-splitter,^1^^,^^2^^,^^14^ the Mirau configuration offers little opportunity of chromatic dispersion mismatch between the two arms of the interferometer. The use of silicone oil with a refractive index of (n=1.4) limits the chromatic dispersion mismatch that may occur during the depth scan into the skin tissues to maintain the short temporal coherence resulting from the large optical bandwidth (FWHM=120±5  nm, centered at 700 nm, see Sec. 4.1).^16^ The presence of silicone oil also ensures that the focal and coherence planes remain superimposed during the depth scan to avoid defocus.^16^

### 2.3 Image Acquisition and Display

**Fig. 3:**
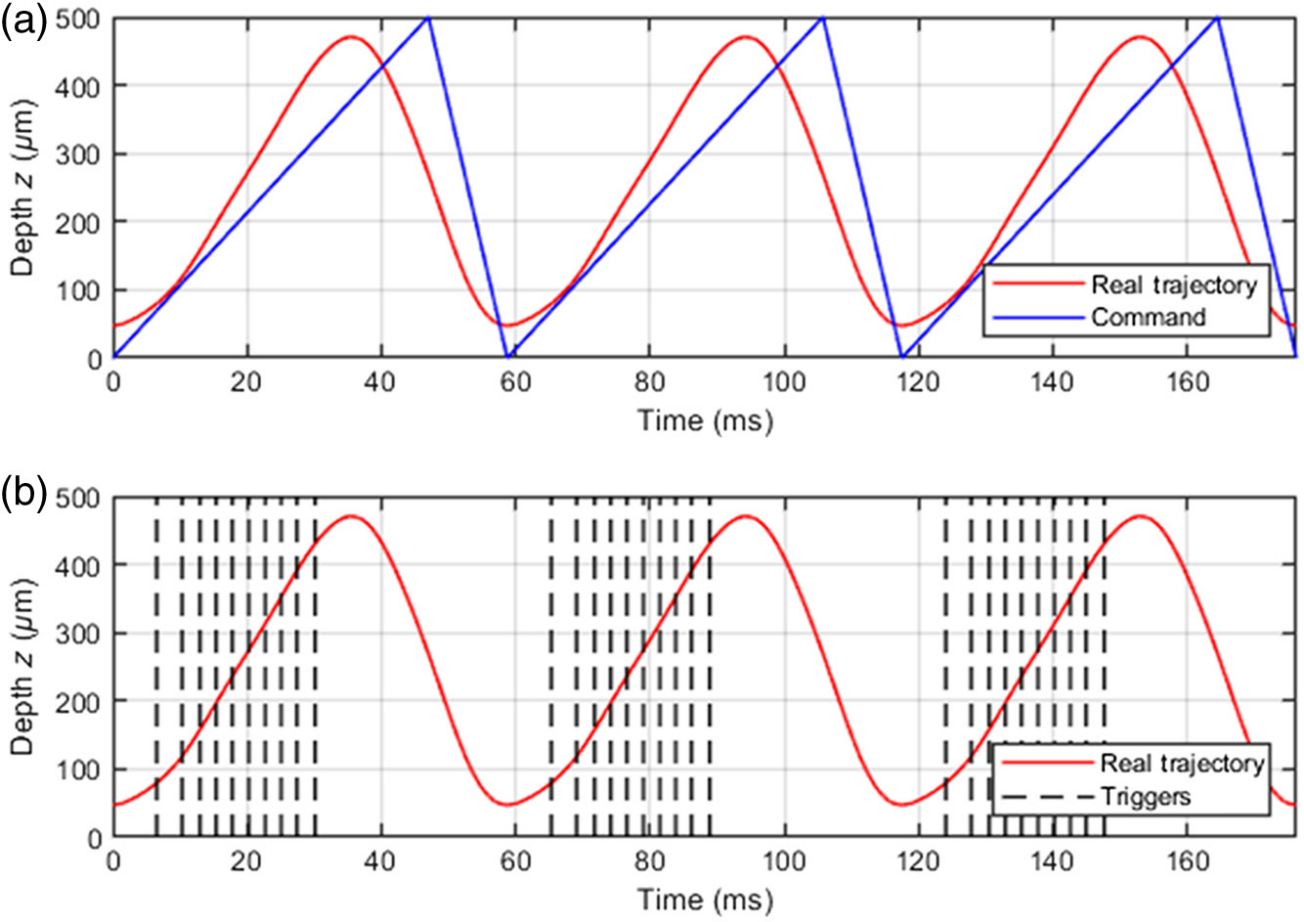
(a) Command sent to the PZT and its real trajectory (depth versus time). (b) Illustration of the triggering of the camera at a variable frequency so that the displacement between successive acquisitions is constant. The vertical dashed lines indicate certain instants of the camera triggering. The camera is triggered only during the positive ramp of the PZT trajectory.

A B-scan is obtained by scanning the line of light in the axial direction (z). This is achieved by translating the whole Mirau interferometer in this direction using the PZT stage (see Figs. 1 and 2). The command sent to the PZT stage and its real trajectory are shown in Fig. 3(a). The command is an asymmetrical triangular waveform repeated periodically at the frequency of 17 Hz with a duty cycle of 80%. Due to inertia, the trajectory of the PZT stage differs from the control, approaching a sinusoidal oscillation. To compensate for the nonlinearity of the trajectory, the camera is triggered at moments such that the displacement between two successive acquisitions by the camera generates a constant phase shift of ϕ=π/2 in the interferometer, as required in the fringe envelope detection algorithm used (Larkin algorithm).^17^ The displacement of the PZT stage between successive acquisitions is then approximately δ=ϕλ/4πn=λ/8n=62  nm, λ=700  nm being the mean detected wavelength and n=1.4 the refractive index of the sample. The triggers to be sent to the camera are determined from the measurement of the trajectory of the PZT stage. They are produced by the electrical generator of a multifunction instrument (Analog Discovery 2). At midstroke, the maximum slope of the trajectory imposes a camera frequency of 235 kHz. The camera frequency is progressively reduced on both sides of the midstroke until the value of 126 kHz is reached at the ends of the stroke where the slope is minimal [see Fig. 3(b)]. The camera exposure is kept constant, regardless of the camera frequency, at 2.33  μs (the maximum camera exposure at 235 kHz). This method is applied to achieve a linear depth scan of 350  μm. A two-dimensional (2D) interferometric image, without distortion, is constructed by stacking 5600 lines acquired during each period of the PZT oscillation. The camera sends its data to a field-programmable gate array (FPGA) that computes a B-scan from the interferometric image using a five-frames fringe envelope detection algorithm (Larkin algorithm).^1^^,^^17^ The B-scans are displayed, with appropriate adjustment of the contrast, at 17 fps. The image acquisition speed is limited by the camera acquisition speed and not by the PZT response nor by the power of the light source. Finely controlling the camera frequency allows for a gain in speed of a factor 1.4 compared with the previously reported Mirau-based LC-OCT device that operated at 12 fps.^15^ In this previous setup, the fixed camera frequency imposed a linear depth scan, which limited the oscillation frequency of the PZT to maintain a depth scan with sufficient amplitude. The gain in speed may seem modest considering the almost three times faster camera used here.^15^ This is mainly due to the deformation of the PZT stage trajectory relative to the command. It can be seen in Fig. 3 that the duration during which the camera performs its acquisitions to scan the depth over 350  μm is shorter than the duration for a trajectory that would follow the command (∼29  ms instead of ∼33  ms). Moreover, the higher the scanning speed is, the steeper the maximum slope of the trajectory (at midstroke) relative to the slope of the ramp command is. The increase of the scanning speed thus requires a more than proportional increase in the camera frequency. In addition, the shorter mean wavelength (700 nm here instead of 800 nm in Ref. 15) requires a smaller step between two successive camera acquisitions (62 nm instead of 71 nm). For the same stroke, the camera must therefore perform more acquisitions.

A 3D image can be obtained by stacking several B-scans. A low-frequency electric generator sends to the galvanometer mirror an asymmetrical triangular electrical voltage that is repeated periodically. This allows for scanning the illumination line linearly in the y direction. The offset and amplitude of the electrical signal are empirically adjusted to make the line of light scan the reference mirror (600  μm scanned for a mirror width of 620  μm), and its oscillation frequency (13.4 mHz with a duty cycle of 95%) is adapted to get a 0.5-μm step between adjacent B-scans. The low-frequency generator is triggered by the FPGA to synchronize the lateral scan with the PZT oscillation and the camera acquisitions. The acquisition of a 3D stack lasts ∼1  min. Once recorded, the 3D stack is postprocessed using MATLAB software. Each 2D interferometric image is processed using the Larkin algorithm to extract the fringe envelope.^1^ The resulting 3D stack is properly rescaled, smoothed with a Gaussian filter, and contrast-adjusted for correct viewing. The size in voxels of the final 3D image is 1880×1200×700, corresponding to an imaged volume of 940  μm×600  μm×350  μm (x×y×z). The sampling step in the final 3D image is thus 0.5  μm in each direction.

## 3 Mirau Interferometer Optimization

### 3.1 Beam-Splitter Reflectivity

The reflectivity of the beam-splitter in the Mirau interferometer is a parameter that can be optimized to maximize the SNR in the LC-OCT images. Neglecting the detection of low reflections on the optical surfaces in the device, the intensities of the reference and sample waves on the sensor of the camera are written, respectively, as

$$I_{\mathrm{Ref}}=I_{0}R_{\mathrm{BS}}^{2}R_{\mathrm{Ref}},$$ (1)

and

$$I_{S}=I_{0}(1-R_{\mathrm{BS}}{)}^{2}R_{S},$$ (2)

where $I_{0}$ is the incident optical intensity, $R_{\mathrm{BS}}$ is the reflectivity of the beam-splitter, and $R_{\mathrm{Ref}}$ is the reflectivity of the reference mirror.  The reflectivity of the sample,$\;R_{S}$, is considered to be the sum of $R_{S,\mathrm{coh}}$ and $R_{S,\mathrm{inc}}$ corresponding to the reflection of light that is coherent and incoherent, respectively, with light reflected by the reference mirror. The intensity of the sample wave is thus written as

$$I_{S}=I_{S,\mathrm{coh}}+I_{S,\mathrm{inc}}=I_{0}(1-R_{\mathrm{BS}}{)}^{2}(\;R_{S,\mathrm{coh}}+\;R_{S,\mathrm{inc}}).$$ (3)

The signal in an LC-OCT image, given by a “coherent” reflector of reflectivity $R_{S,\mathrm{coh}}$, is proportional to the square of the interferometric signal, i.e., to $I_{S,\mathrm{coh}}\times I_{\mathrm{Ref}}$. Assuming a shot-noise limited detection, the background noise in an LC-OCT image (mean signal measured in the absence of “coherent” reflector) is proportional to $I_{S,\mathrm{inc}}+I_{\mathrm{Ref}}$. The SNR, defined as the ratio between the signal and the background noise, is therefore

$$\mathrm{SNR}\propto \frac{R_{S,\mathrm{coh}}R_{\mathrm{Ref}}R_{\mathrm{BS}}^{2}(1-R_{\mathrm{BS}}{)}^{2}}{R_{S,\mathrm{inc}}(1-R_{\mathrm{BS}}{)}^{2}+R_{\mathrm{Ref}}R_{\mathrm{BS}}^{2}}.$$ (4)

The value of $R_{\mathrm{BS}}$ that maximizes the SNR can be calculated by taking the derivative of Eq. (4) with respect to $R_{\mathrm{BS}}$. This leads to the resolution of a cubic equation, with a real root of

$$R_{\mathrm{BS}}=(1+R_{S,\mathrm{inc}}/R_{\mathrm{Ref}}{)}^{-1}((R_{S,\mathrm{inc}}/R_{\mathrm{Ref}}{)}^{1/3}-(R_{S,\mathrm{inc}}/R_{\mathrm{Ref}}{)}^{2/3}+R_{S,\mathrm{inc}}/R_{\mathrm{Ref}}).$$ (5)

If $R_{S,\mathrm{inc}}\ll 1$, an approximate expression of the optimal value of $R_{\mathrm{BS}}$ is

$$R_{\mathrm{BS}}∼(R_{S,\mathrm{inc}}/R_{\mathrm{Ref}}{)}^{1/3}.$$ (6)

**Fig. 4:**
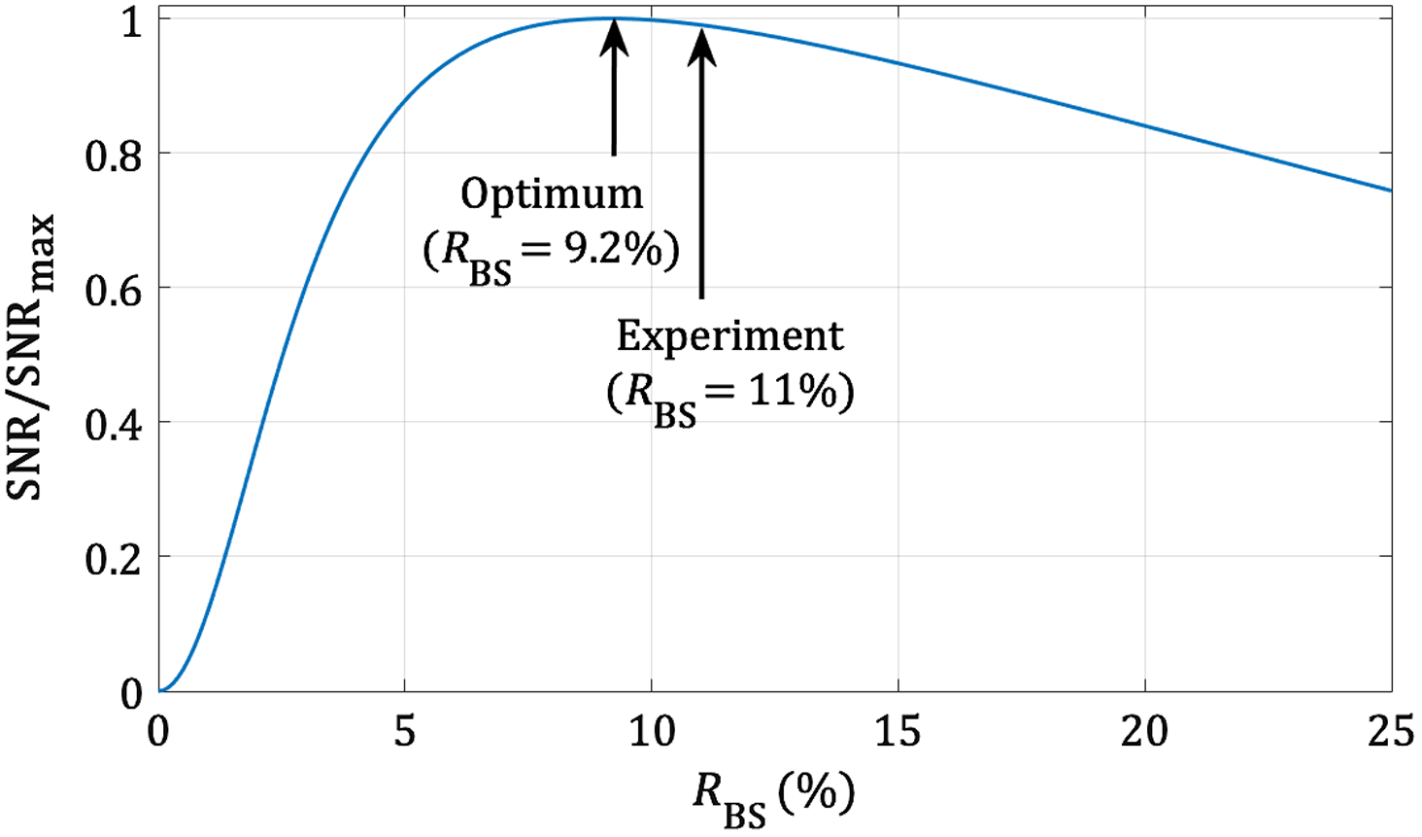
Normalized theoretical SNR versus reflectivity of the beam-splitter ($R_{\mathrm{BS}}$). Simulation based on Eq. (4), with the following parameters: $R_{\mathrm{Ref}}=96\%$ and $R_{S,\mathrm{inc}}=0.1\%$. SNR is maximum for $R_{\mathrm{BS}}=\;9.2\%$. $\mathrm{SNR}/{\mathrm{SNR}}_{\max}=0.99$ for $R_{\mathrm{BS}}=11\%$ (experiment).

The typical reflectivity of skin tissues was measured as $R_{S}∼0.1\%$ by comparison with the reflectivity of the interface glass/air (reflectivity of 3.4%). Due to the low temporal coherence of the light source, most of the light backscattered by the sample is not coherent with light reflected by the reference mirror, which implies that $R_{S}∼R_{S,\mathrm{inc}}\ll 1$. The theoretical value of the optimal reflectivity of the beam-splitter is $R_{\mathrm{BS}}=\;9.2\%$. The thickness of the a-SiH reflective layer of the beam-splitter was adjusted to obtain a reflectivity close to the optimal theoretical value. With an effective reflectivity measured at $R_{\text{BS}}=11\%$, the theoretical SNR has a value multiplied by a factor of 0.99 compared with its maximum (see Fig. 4). The beam-splitter of the Mirau-based LC-OCT device can therefore be considered to be optimized.

### 3.2 Beam Obscuration

**Fig. 5:**
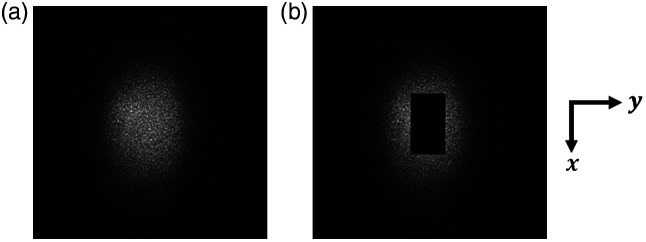
Estimation of the amount of light lost due to the central obscuration by the reference mirror of the Mirau interferometer. Each image is the light exiting the last diopter of the microscope objective after putting a diffuser on it (a) without or (b) with a numerical mask of dimensions 1100  μm×620  μm applied. A total of 31% of light is lost with the mask.

The presence of a mirror in the optical path of the Mirau interferometer leads to a loss of light, which is a drawback of the Mirau configuration compared with the Linnik configuration.^18^^–^^20^ In the previously reported Mirau-based LC-OCT device,^15^ the reference mirror had a rectangular shape with a length of 2 mm and a width of 100μm. The loss of light was weak (8%). In the reported device designed for 3D imaging, the lateral scan of the illumination line requires a reference mirror with a width that is at least equal to the lateral field of view (in the y direction). A compromise between the lateral field of view and the amount of light loss has to be made. The beam intensity distribution was measured on the last diopter of the microscope objective by putting a diffuser on it and taking an image of the diffuser using an area camera with a calibrated photometric response. Numerical application of rectangular masks on the images simulating the presence of the reference mirror allowed for quantifying the light loss. Finally, a reference mirror with dimensions of 1100  μm×620  μm was considered a good compromise. The loss of light is then 31% (see Fig. 5). This choice was made considering the available power of the light source used here. Indeed, using a less powerful light source would result in reducing the size of the reference mirror, and thus of the field of view, or slowing down the acquisition speed. It should be mentioned that the light loss varies when the beam is scanned laterally. This is due to the fact that the plane of the reference mirror is not conjugated to the galvanometric scanner mirror. The light loss, measured at 31% at the center, reaches 54% at the maximum scan angle. As a result, the SNR of B-scans decreases as the scan angle increases. The resulting degradation of image quality is noticeable but not significant.

## 4 Imaging Performance

### 4.1 Spatial Resolution

**Fig. 6:**
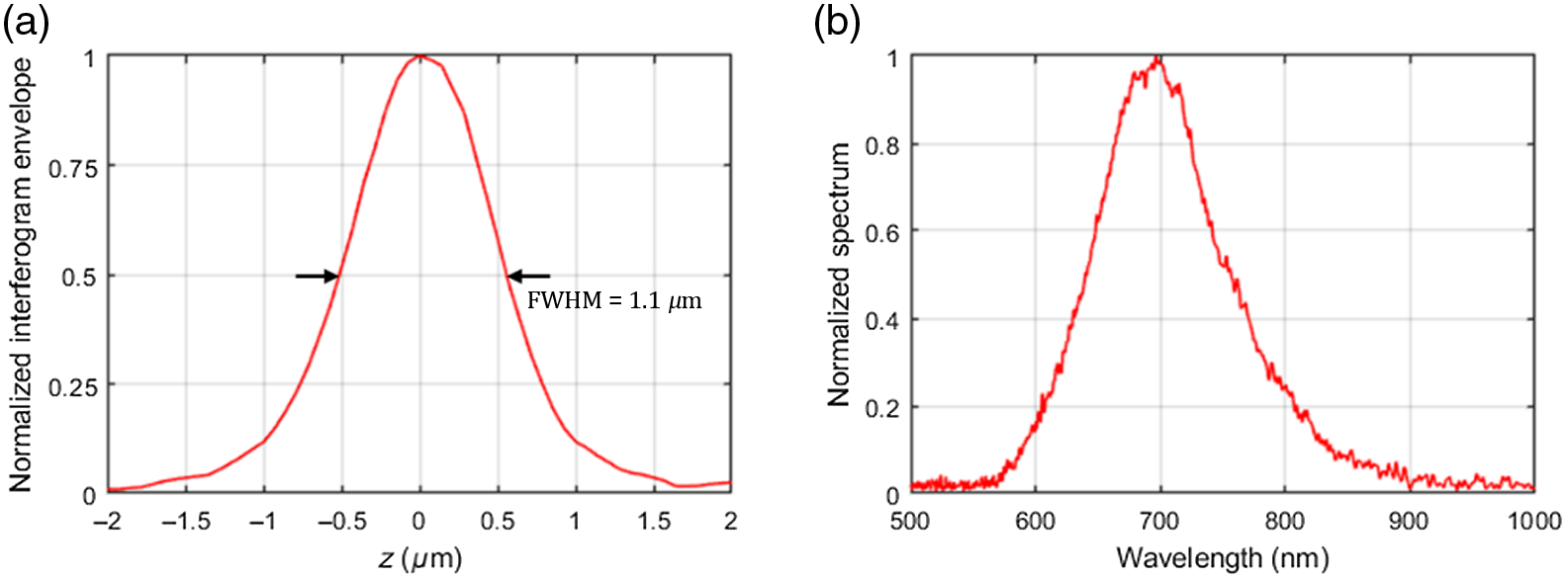
(a) Normalized envelope of the interferogram generated by the reflection on a glass-air surface and obtained using the Larkin algorithm. The axial resolution, defined as the FWHM of the envelope, is equal to 1.1  μm. (b) Spectrum of light detected by the camera and derived from the Fourier transform of the interferogram. The center of mass is found at 700±10  nm, and the width (FWHM) is at  120±5  nm.

An interferogram was recorded by imaging the air-glass interface against which the sample is pressed, with the sample removed. The axial resolution, defined as the full-width-at-half-maximum (FWHM) of the envelope of the interferogram, was measured at 1.1  μm [see Fig. 6(a)].

The spectrum of the light detected by the camera can be derived through the calculation of the Fourier transform of the interferogram [see Fig. 6(b)]. The mean wavelength, defined as the center of mass of the spectrum, is 700±10  nm. The full width at half maximum of the spectrum is 120±5  nm.

**Fig. 7:**
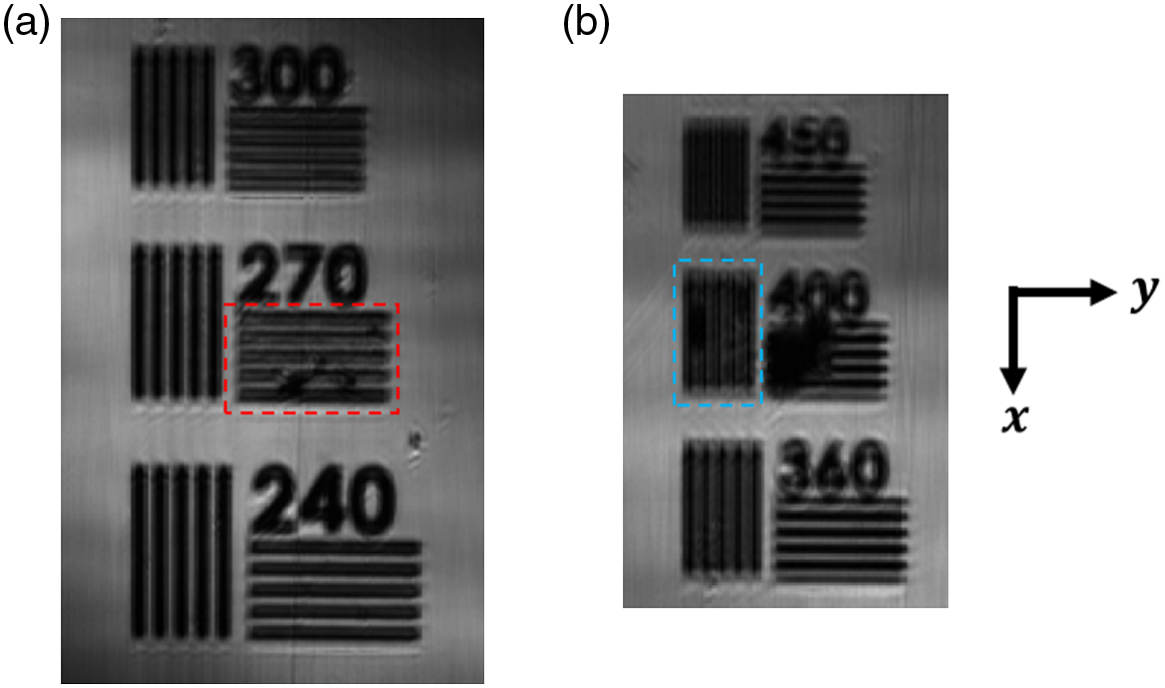
Measurement of the lateral resolution. (a) and (b) Patterns of a resolution target imaged to determine the lateral resolution in the direction of the illumination line (x) and in the direction orthogonal to the illumination line (y), respectively. The measured resolutions are (a) 270 lp/mm and (b) 400 lp/mm, corresponding to 1.9 and 1.3  μm, respectively. The difference in resolutions between the two directions is attributed to the illumination conditions: along a line in the x direction and “point-scanning” in the y direction.

The lateral resolution was measured by imaging a high-resolution target (Target #37-539, Edmund Optics). A stack of 2D interferometric images was acquired, processed using the Larkin algorithm,^1^^,^^17^ and resized to get a 3D image. No more image processing was done. An *en face* image of the target was extracted from the 3D image (see Fig. 7). To work in similar conditions to those during the acquisition of images of skin, the output power of the light source was the same, but a neutral optical density was placed in the beam path to avoid saturating the sensor. The lateral resolution is defined as the spatial frequency or the size of the last pattern for which the number of dark bars can be counted correctly and without ambiguity. The lateral resolution is estimated at 270 lp/mm in the direction of the line of light (x) and 400 lp/mm in the orthogonal direction (y), corresponding to 1.9 and 1.3  μm, in the x and y directions, respectively. Even if smaller patterns can be seen with a good contrast, either counting the bars becomes ambiguous or the patterns are aliased. The lateral resolutions in the direction of the line of light (x) and in the orthogonal direction (y) differ because the illumination conditions in each direction are different. Illumination with spatially coherent light along a line causes edge-ringing artifacts in the direction of this line (x direction).^21^^,^^22^ The confocal effect, i.e., “point” illumination and “point” detection, in the direction of the lateral scan (*y* direction) strongly mitigates these artifacts and leads to better resolution.^21^^–^^23^ The measured resolution is significantly lower than the theoretical diffraction-limited resolution of the microscope objective illuminated at the wavelength of 700 nm by a Gaussian beam with a $1/e^{2}$ diameter of 6.4 mm: full-width-at-half-maximum (FWHM) of the point-spread function (PSF) ∼0.8  μm. The degradation of the resolution is attributed to optical aberrations caused by the presence of three glass plates (total thickness 1.17 mm) above the sample, the use of oil rather than water as an immersion medium, and the central obscuration of light by the reference mirror of the Mirau interferometer (see Fig. 2).

### 4.2 Skin Imaging

To demonstrate the performance of the Mirau-based LC-OCT device for skin imaging, 3D images of healthy skin from a 26-year-old man were acquired. A drop of paraffin oil was put on the skin before it was pressed against the glass window for imaging. Paraffin oil between the skin surface and the glass window provides index matching and thus minimizes the reflection of light at the interfaces. The skin was illuminated with a power of 55 mW distributed according to a Gaussian of 1150-μm width (FWHM) along the line of light (x direction) and a Gaussian of 1.3-μm width (FWHM) in the direction orthogonal to the line of light (y direction). The maximal exposure time of the skin for the acquisition of a line by the camera was 1/126  kHz∼8  μs. The radiant exposure (fluence) on the skin was evaluated as $55.10^{-3}\times 8.10^{-6}/(1.3.10^{-6}\times 1150.10^{-6})$ $∼290\;J.m^{-2}$. In the spectral range of the experiment, the international standard IEC 60825-1:2014 indicates a maximum permissible exposure (MPE) of $584\;J.m^{-2}$.^24^

**Fig. 8:**
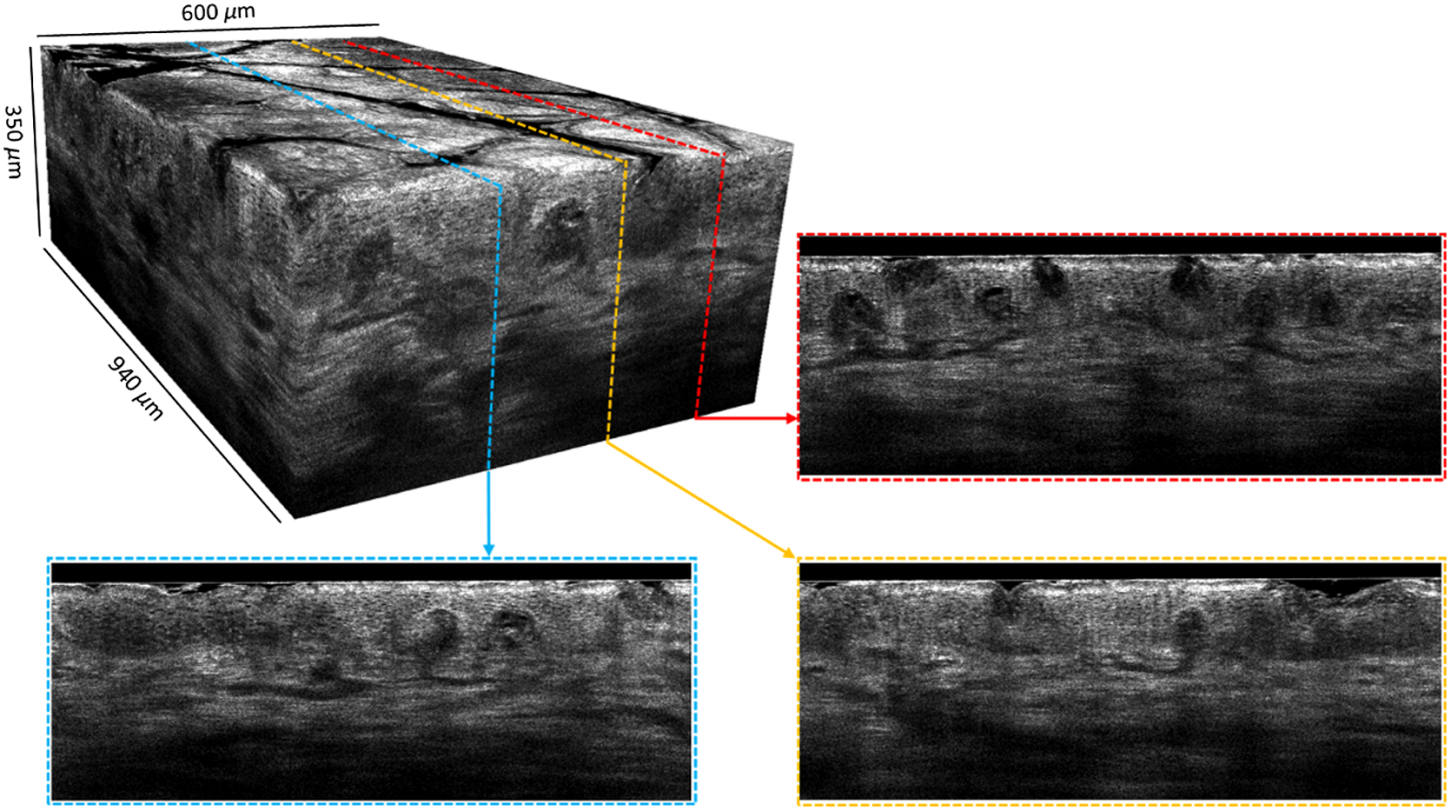
Example of a 3D image of skin on the back of the hand. Several vertical sections are also shown. The size of the volume is 940  μm×600  μm×350  μm (x×y×z). The 3D image is made of 1200 vertical sections (B-scans) acquired at 17 fps, separated by 0.5  μm from one another.

**Fig. 9:**
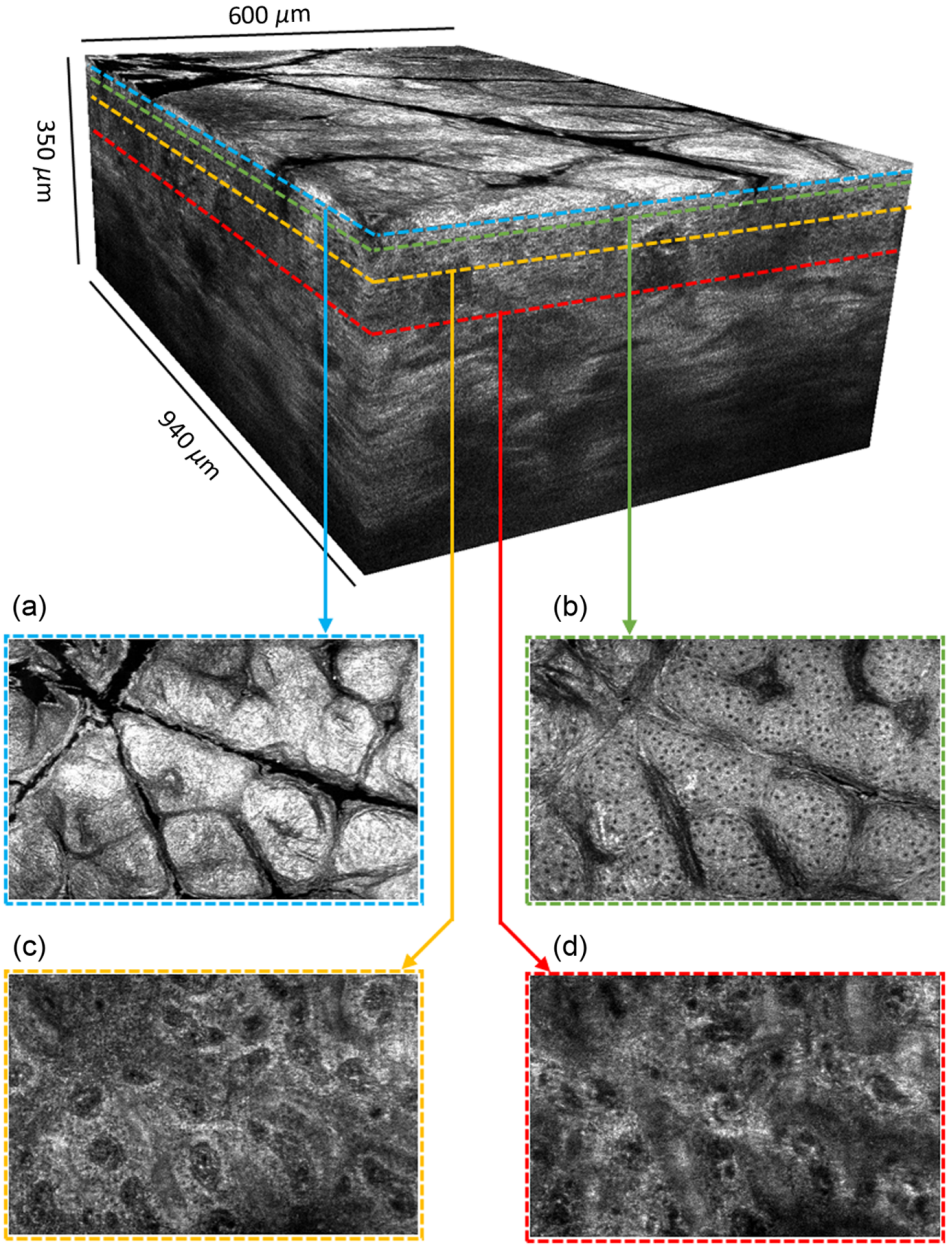
Example of a 3D image of healthy skin on the back of the hand. Several horizontal slices illustrating the layers of human skin are also shown, from top to bottom of the sample: (a) stratum corneum, (b) stratum spinosum, (c) stratum basale, and (d) papillary dermis.

**Fig. 10:**
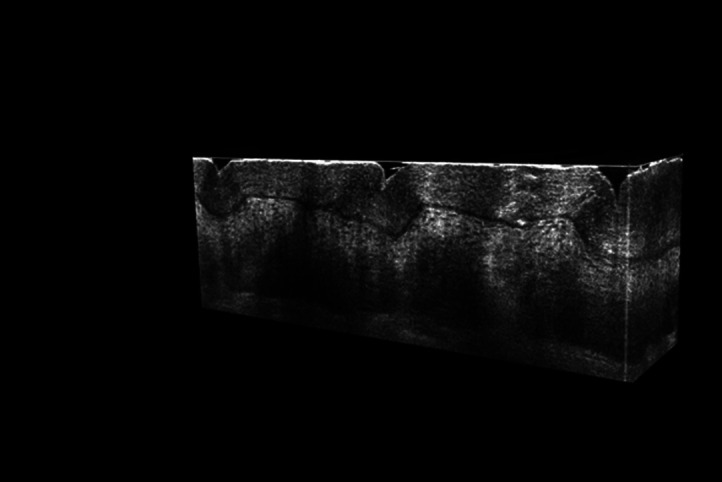
Navigation through a 3D image of healthy skin on the side of a finger (Video 1, mp4, 16.4 MB [URL: https://doi.org/10.1117/1.JBO.27.8.086002.1]).

All of the 3D images were acquired in approximately one minute and then postprocessed using MATLAB software. 3D Gaussian filtering with a standard deviation of 0.5 pixel was applied to the images. The light intensity along the line field was Gaussian, so image processing was applied to numerically flatten the illumination in the acquired images. However, a degradation of the SNR is unavoidable and is slightly visible at the edges of the LC-OCT images. The software 3D Slicer was used to display the images. Figures 8 and 9 show vertical and horizontal sections, extracted from a 3D image acquired on the back of the hand. The epidermis and the dermis, separated by the dermal-epidermal junction, can be clearly distinguished. The nuclei of keratinocyte cells in the epidermis are resolved and appear as black spots. Collagen fibers and blood vessels are visible in the dermis. Figure 10 is a video that illustrates the navigation through a 3D image acquired on the side of a finger. Compared with the back of the hand, the stratum corneum in the finger region is thicker. A helical sweat duct is visible.

## 5 Conclusion

We have presented an improved LC-OCT device based on a Mirau interferometer that is able to acquire and display B-scans at 17 fps. 3D images with a quasi-isotropic resolution of ∼1.5  μm over a field of 940  μm×600  μm×350  μm (x×y×z) were obtained by stacking B-scans acquired at different positions. 3D imaging of human skin at cellular resolution, *in vivo*, was demonstrated with the device.

The acquisition rate of the B-scans, at 17 fps, is unprecedented in LC-OCT. Compared with the conventional LC-OCT devices based on a Linnik interferometer, the reported Mirau-based LC-OCT device acquired B-scans about two times faster. A higher operation speed was achieved by dynamic adjustment of the camera frequency during the depth scan to compensate for the nonlinearity of the trajectory and using a faster camera and a more powerful light source. The reduction of the mass to be oscillated also contributed to making a higher speed possible.

The fastest frequency-domain OCT devices reported to date can acquire A-scans at rates up to ∼300  kHz.^25^^,^^26^ and even up to ∼20  GHz by acquiring 4 A-scans in parallel.^27^ For comparison with the LC-OCT device presented here, they produce B-scans made of 2048 A-scans at a rate up to $∼10^{4}\;\mathrm{fps}$.^27^ However, frequency-domain OCT produces images with significantly lower lateral resolution because dynamic focusing cannot be applied (∼15× lower in Ref. 27). The ability to continuously adjust the focus to obtain B-scans with a lateral resolution of ∼1.5  μm, while producing these images at 17 frames per second, is the major interest of the proposed imaging technique.

The acquisition time of a 3D image (1 min) is too long for the technique to be easily adopted clinically. However, a significant reduction of this time should be possible by exploiting the round trips of a sinusoidal trajectory of the PZT stage (only the one-way trips of a quasi-sinusoidal trajectory were used here). This should be possible by adopting the principle of acquisition with a variable frequency camera demonstrated in this paper.

The reflectivity of the beam-splitter in the Mirau interferometer was optimized to maximize the image SNR. Compared with the last reported Linnik-based LC-OCT device, however, an illumination of the skin about 10 times more powerful is required. This is a result of the beam obscuration by the reference mirror in the Mirau interferometer, the longer focal length of the tube lens and some vignetting by this lens, and especially the shorter camera exposure time of ∼2.33  μs (i.e. ∼3  times shorter). This exposure time corresponds to its maximum value when the camera operates at its maximum effective frequency of 235 kHz required to achieve an operation speed of 17 fps. However, the illumination of the skin is still within the international maximum permissible exposure standard.

Finally, with potential advantages in terms of compactness and weight compared with the Linnik-based LC-OCT devices, a Mirau-based device could easily be integrated into a smaller and lighter handheld probe for use by dermatologists in their daily medical practice.
